# SapTrap Assembly of *Caenorhabditis elegans* MosSCI Transgene Vectors

**DOI:** 10.1534/g3.119.400822

**Published:** 2019-12-17

**Authors:** Xintao Fan, Sasha De Henau, Julia Feinstein, Stephanie I. Miller, Bingjie Han, Christian Frøkjær-Jensen, Erik E. Griffin

**Affiliations:** * Department of Biological Sciences, Dartmouth College, Hanover NH 03755,; † Center for Molecular Medicine, Molecular Cancer Research, University Medical Center Utrecht, 3584 CG Utrecht, The Netherlands, and; ‡ King Abdullah University of Science and Technology (KAUST), Biological and Environmental Science and Engineering Division (BESE), KAUST Environmental Epigenetics Program (KEEP), Thuwal 23955-6900, Saudi Arabia

**Keywords:** MosSCI, *C. elegans*, mitochondria, endoplasmic reticulum, SapTrap

## Abstract

The Mos1-mediated Single-Copy Insertion (MosSCI) method is widely used to establish stable *Caenorhabditis elegans* transgenic strains. Cloning MosSCI targeting plasmids can be cumbersome because it requires assembling multiple genetic elements including a promoter, a 3′UTR and gene fragments. Recently, Schwartz and Jorgensen developed the SapTrap method for the one-step assembly of plasmids containing components of the CRISPR/Cas9 system for *C. elegans*. Here, we report on the adaptation of the SapTrap method for the efficient and modular assembly of a promoter, 3′UTR and either 2 or 3 gene fragments in a MosSCI targeting vector in a single reaction. We generated a toolkit that includes several fluorescent tags, components of the ePDZ/LOV optogenetic system and regulatory elements that control gene expression in the *C. elegans* germline. As a proof of principle, we generated a collection of strains that fluorescently label the endoplasmic reticulum and mitochondria in the hermaphrodite germline and that enable the light-stimulated recruitment of mitochondria to centrosomes in the one-cell worm embryo. The method described here offers a flexible and efficient method for assembly of custom MosSCI targeting vectors.

The rich toolbox of techniques available to manipulate gene expression in *C. elegans* is a major attraction of this model organism. Several approaches have been developed to introduce transgenes and to induce efficient CRISPR/Cas9 mediated gene editing (Nance and Frøkjær-Jensen 2019). The Mos1-mediated Single-Copy Insertion (MosSCI) method has been widely adopted to introduce transgenes in *C. elegans* because single-copy transgenes are integrated at defined chromosomal positions, thereby mitigating potential concerns of transgene integration at random positions (Frøkjær-Jensen *et al.* 2008; Frøkjær-Jensen *et al.* 2012; Frøkjær-Jensen *et al.* 2014). MosSCI transgene integration results from homologous recombination between a MosSCI targeting vector containing the transgene construct and one of the safe-harbor integration sites that have been engineered at defined positions in the genome.

Transgenes typically include multiple genetic elements including a promoter, one or more gene fragments and a 3′UTR. A number of strategies can be used to assemble these elements together including traditional restriction enzyme cloning, Gateway cloning (Hartley *et al.* 2000), *in vivo* recombineering (Philip *et al.* 2019) or Gibson cloning (Gibson *et al.* 2009). Each of these strategies has both advantages and disadvantages. For example, Gateway cloning allows the efficient modular “mix and match” cloning of large collections of promoter, ORF and 3′UTR cassettes (Brasch *et al.* 2004; Dupuy *et al.* 2004; Mangone *et al.* 2010; Zeiser *et al.* 2011). However, Gateway cloning can be expensive due to the required use of proprietary enzyme mixes and leaves ∼25 base pair *att* recombination site “scars” at each cassette junction. In contrast, Gibson cloning allows the efficient, “scar-free” assembly of multiple gene fragments but does not allow the “mix and match” cloning of existing cassettes, making this approach laborious if many constructs are needed.

Schwartz and Jorgensen recently developed the SapTrap method for efficient, modular and single step assembly of CRISPR/Cas9 vectors for *C. elegans* (Schwartz and Jorgensen 2016). The SapTrap method is based on the Golden Gate cloning technique (Engler *et al.* 2008) and takes advantage of the SapI type II restriction enzyme, which cuts DNA at defined positions adjacent to its recognition sequence to generate three-base 5′ overhangs. By designing SapI restriction fragments with complementary overhangs, multiple fragments can be assembled together in a defined order in a single digestion and ligation reaction. In this study, we report on the adaptation of the SapTrap system for the efficient, inexpensive, modular, and “scar-free” assembly of transgenes in a MosSCI targeting vector. We have developed a toolkit for expression of transgenes in the *C. elegans* germline, including a collection of cassettes containing tags for fluorescence imaging and for the ePDZ/LOV optogenetic system (Strickland *et al.* 2012; Fielmich *et al.* 2018). As a proof of principle, we have used this system to generate a collection of mitochondrial and endoplasmic reticulum reporter strains and a strain in which light induces the transport of mitochondria to centrosomes in the one-cell worm embryo.

## Materials and Methods

### C. elegans

*C. elegans* hermaphrodite strains were maintained at either 20° or 25° on Nematode Growth Medium (NGM) plates containing 3 g/L NaCl, 2.5 g/L peptone and 17 g/L agar supplemented with 1 mM CaCl_2_, 1 mM MgSO_4_, 1 mM KPO_4_ and 5 mg/L cholesterol with *E. coli* OP50 as a source of food. All strains used in this study are listed in Table 1.

**Table 1:** Strains used in this study

Strain	Genotype	Construction	Reference:
EG8078	*oxTi185 I*; *unc-119(ed3) III*		Frøkjær-Jensen *et al.* (2014)
EG8079	*oxTi179 II*; *unc-119(ed3) III*		Frøkjær-Jensen *et al.* (2014)
EGD329	*egxSi126 [mex-5p*::*hsp-3(aa 1-19)*::*halotag*::hdel::*pie-1 3′UTR + unc119(+)] I*; *unc-119(ed3) III*	Injected pJF13 into EG8078	This study
EGD412	*egxSi136 [mex-5p*::*tomm-20*::*halotag*::*pie-1 3′UTR + unc119(+)] II*; *unc-119(ed3) III*	Injected pJF17 into EG8079	This study
EGD496	*egxSi117 [pmex-5p*::*npp-20*::*gfp;;pie-1 3′UTR + unc119(+)] I*; *unc-119(ed3) III*	Injected pXF253 into EG8078	This study
EGD497	*egxSi118 [mex-5p*::*npp-20*::*halotag*::*pie-1 3′UTR + unc119(+)] II*; *unc-119 (ed3) III*	Injected pXF255 into EG8079	This study
EGD549	*egxSi144 [mex-5p*::*cox-4*::*halotag*::*pie-1 3′UTR + unc119(+)] II*; *unc-119 (ed3) III*	Injected pXF266 into EG8079	This study
EGD565	*egxSi145 [mex-5p*::*hsp-3(aa 1-19)*::*halotag*::hdel::*pie-1 3′UTR + unc119(+)] II*; *unc-119 (ed3) III*	Injected pJF13 into EG8079	This study
EGD623	*egxSi152 [mex-5p*::*tomm-20*::*gfp*::*pie-1 3′UTR + unc119(+)] II*; *unc-119(ed3) III*	Injected pSM16 into EG8079	This study
EGD629	*egxSi155 [mex-5p*::*tomm-20*::*mkate2*::*pie-1 3′UTR + unc119(+)] II*; *unc-119(ed3) III*	Injected pSM20 into EG8079	This study
EGD631	*egxSi157 [mex-5p*::*tomm-20*::*dendra2*::*pie-1 3′UTR + unc119(+)] II*; *unc-119(ed3) III*	Injected pSM17 into EG8079	This study
EGD633	*egxSi159 [mex-5p*::*tomm-20*::*mscarlet*::*pie-1 3′UTR + unc119(+)] II*; *unc-119(ed3) III*	Injected pSM22 into EG8079	This study
EGD615	*cox-4(zu476[cox-4*::*eGFP*::*3XFLAG]) I*; *egxSi136 [mex-5p*::*tomm-20*::*halotag*::*pie-1 3′UTR + unc119(+)] II*; *unc-119(ed3?) III*	Crossed EGD412 and JJ2586	This study
JJ2586	*cox-4(zu476[cox-4*::*eGFP*::*3XFLAG]) I*		Raiders *et al.* 2018
TBD307	*dhc-1(he255[epdz*::*mcherry*::*dhc-1]) I*; *utdSi51(mex-5p*::*tomm-20(aa 1-55)*::*halotag*::*lov*::*tbb-2 3′UTR + unc119(+)) II*	Injected pSDH68 into EG8079. Crossed to SV2095.	This study
SV2095	*dhc-1(he255[epdz*::*mcherry*::*dhc-1]) I*; *ruls57[pie-1p*::*gfp*::*tbb-2 + unc119(+)] V*		Fielmich *et al.* 2018

### Cloning

To generate the expression vector pXF87, the two SapI restriction sites in pCFJ350 (Frøkjær-Jensen *et al.* 2012) were mutated using Q5 Site-Directed Mutagenesis (New England Biolabs (NEB)) with the oligo pairs XF30F/XF30R and XF31F/XF31R. In addition, the annealed oligos Eg717 and Eg718 were cloned between the XhoI and SpeI sites of pCFJ350.

HaloTag and ceGFP containing PATC-rich endogenous introns were generated in several steps. First, genes were designed *in silico* to minimize germline silencing and increase expression by codon adaptation (Redemann *et al.* 2011), removal of homology to piRNAs (Batista *et al.* 2008), and inclusion of a short endogenous intron from *rpl-18* and four synthetic introns (Okkema *et al.* 1993) using the freely available gene editor ApE (M. Wayne Davis, https://jorgensen.biology.utah.edu/wayned/ape/). Second, the synthetic genes were synthesized as gBlocks (IDT), cloned into a plasmid, and sequence verified. Third, PATC-rich introns from a gene that is resistant to germline silencing, *smu-1* (Spike *et al.* 2001), were introduced into the synthetic genes by Golden Gate cloning as described previously (Frøkjær-Jensen *et al.* 2016). Finally, correct splicing and expression was verified by expression of the synthetic genes with and without PATC-rich introns using an *eft-3* promoter and *tbb-2* 3′UTR.

Donor cassette plasmids numbered pXF, pJF and pSM were generated by cloning PCR products into the pCR BluntII vector backbone using the Zero Blunt Topo system (Thermo Fisher Scientific). pSDH donor cassette plasmids were cloned by ligating PCR products into pSDH76, a derivative of pCR BluntII containing two XcmI sites that generate T-overhangs following digestion with XcmI. pXF87 and all donor plasmids were sequence verified.

To assemble HSP-3 (aa 1-19) into the second cassette of the expression vector pJF13, 10 μM of oligos XF17F and XF17R were gradually cooled from 95° to 25° in a BioRad T1000 thermocycler. Annealed oligos were phosphorylated by T4 polynucleotide kinase (NEB) for two hours at 37°, followed by an enzyme inactivation step at 65° for 20 min. The donor plasmids and primers are listed in Tables 2 and 3, respectively.

**Table 2:** Donor cassette plasmids used in this study

Name	Description
**Cassette 1 for 4-cassette or 5 cassette system (5′-TGG ...... 3′-TAC)**	
pXF121	*mex-5* promoter
pSDH60	*spe-11* promoter
**Cassette 2 for 4-cassette or 5-cassette system (5′-ATG-3′ ...... 3′-CCA-5′)**	
***Tags***	
pXF89	*halotag* (no STOP codon, PATC introns)
pJF5	*gfp* (no STOP codon, PATC introns)
pXF222	*mkate2* (no STOP codon)
pSDH61	*epdz* (no STOP codon)
pSM10	*mscarlet* (no STOP codon)
pSM12	*dendra2* (no STOP codon)
***Genes***	
pJF7	*tomm-20* (no STOP codon)
pSDH50	*tomm-20* (aa 1-55) (no STOP codon)
pXF262	*cox-4* (no STOP codon)
pXF250	*npp-20* (no STOP codon)
**Cassette 3 for 4-cassette system (5′-GGT-3′ ...... 3′-ATT-5′)**	
***Tags***	
pXF88	*halotag* (includes STOP codon, PATC introns)
pJF6	*gfp* (includes STOP codon, PATC introns)
pXF130	*mkate2* (includes STOP codon)
pSM08	*mscarlet* (includes STOP codon)
pSM03	*dendra2* (includes STOP codon)
***ORFs***	
pXF90	*halotag*::hdel (includes STOP codon, PATC introns)
**Cassette 3A for 5-cassette system (5′-GGT-3′ ...... 3′-TGC-5′)**	
***Tags***	
pSDH51	*halotag* (no STOP codon, PATC introns)
pSM04	*mkate2* (no STOP codon)
pSDH57	*mscarlet* (no STOP codon)
**Cassette 3B for 5-cassette system (5′-ACG-3′ ...... 3′-ATT-5′)**	
***Tags***	
pXF276	*lov* domain (includes STOP codon)
pSDH52	*epdz* (includes STOP codon)
pSM05	*mkate2* (includes STOP codon)
**Cassette 4 for 4-cassette or 5-cassette system (5′-TAA-3′ ...... 3′-CAT-5′)**	
pXF85	*pie-1* 3′UTR
pSDH54	*tbb-2* 3′UTR
pSDH66	*unc-54* 3′UTR

**Table 3:** Primers used in this study

Name	Description	Sequence (SAP1 site and Overhang)	Corresponding plasmid
XF32F	*mex-5 promoter* (F)	GCAGCTCTTCG**TGG**ATATCAGTTTTTAAAAAATTA	pXF121
XF32R	*mex-5 promoter* (R)	GCAGCTCTTCG**CAT**TCTCTGTCTGAAACA
JF5F	*tomm-20* (F)	GCAGCTCTTCG**ATG**TCGGACACAATTCTTGG	pJF7
JF5R	*tomm-20* (R)	GCAGCTCTTCG**ACC**CTCCAAGTCGTCGGTGTC	
JF1F	*gfp* (F)	GCAGCTCTTCG**ATG**TCCAAGGTAACACTTAGTTT	pJF5
JF1R	*gfp* (R)	GCAGCTCTTCG**ACC**GCCGCTTCCCTTGTAGAGCTCGTCCAT	
JF2F	*gfp* (F)	GCAGCTCTTCG**GGT**GGAAGCGGCTCCAAGAACACTTAGTTT	pJF6
JF2R	*gfp* (R)	GCAGCTCTTCG**TTA**CTTGTAGAGCTCGTCCAT	
XF17F	*hsp-3 (1-19aa)* (F)	**ATG**AAGACCTTATTCTTGTTGGGCTTGATCGCCCTATCCGCCGTCAGTGTCTACTGC	
XF17R	*hsp-3 (1-19aa)* (R)	**ACC**GCAGTAGACACTGACGGCGGATAGGGCGATCAAGCCCAACAAGAATAAGGTCTT
spe-11(SAP C1) F	*spe-11 promoter* (F)	GCAGCTCTTCG**TGG**GTCGACAGAACATTTTTCCGT	pSDH60
spe-11(SAP C1) R	*spe-11 promoter* (R)	GCAGCTCTTCG**CAT**TTTATCTAGTCGGTTTGCGA	
XF24F	*halotag* (F)	GCAGCTCTTCG**ATG**GCCGAGGTAACACTTAGTTTTTGT	pXF89
XF24R	*halotag* (R)	GCAGCTCTTCG**ACC**GCCGCTTCCTCCGGAGATCTCGAGGGT	
XF63F	*mkate2* (F)	GCAGCTCTTCG**ATG**GTCTCCGAGCTCATTAAAGAAAACA	pXF222
XF63R	*mkate2* (R)	GCAGCTCTTCG**ACC**ACCTCCACCTCCACGGTGTCCGAGCTTGG	
ePDZ (SAP C2) F	*epdz* (F)	GCAGCTCTTCG**ATG**CCAGAGCTCGGATTCTCGAT	pSDH61
ePDZ (SAP C2) R	*epdz* (R)	GCAGCTCTTCG**ACC**AGCTCCCGTCGCGACGGGTGGATCAC	
XF79F	*cox-4* (F)	GCAGCTCTTCG**ATG**ATGCTGCCACGTTTG	pXF262
XF79R	*cox-4* (R)	GCAGCTCTTCG**ACC**CTTCCACTTCTTGTTCTCGTAATC	
XF76F	*npp-20* (F)	GCAGCTCTTCG**ATG**ACCACGGTCCGCCAG	pXF250
XF76R	*npp-20* (R)	GCAGCTCTTCG**ACC**TCTCTGAGCTCCCGGAGCT	
XF23F	*halotag* (F)	GCAGCTCTTCG**GGT**GGAAGCGGCGCCGAGGTAACACTTAGTTTTTGT	pXF88
XF23R	*halotag* (R)	GCAGCTCTTCG**TTA**TCCGGAGATCTCGAGGGT	
XF53F	*mkate2* (F)	GCAGCTCTTCG**GGT**GGAGGTGGAGGTGTCTCCGAGCTCATTAAAGAAAAC	pXF130
XF53R	*mkate2* (R)	GCAGCTCTTCG**TTA**ACGGTGTCCGAGCTTGGA	
XF22F	*halotag*::*hdel* (F)	GCAGCTCTTCG**GGT**GGAAGCGGCGCCGAGGTAACACTTAGTTTTTGT	pXF90
XF22R	*halotag*::*hdel* (R)	GCAGCTCTTCG**TTA**GAGTTCGTCATGTCCGGAGATCTCGAGGGT	
SIM8F	*mscarlet* (F)	GCAGCTCTTCG**ATG**GTCTCCAAGGGCGAGGCA	pSM10
SIM8R	*mscarlet* (R)	GCAGCTCTTCG**ACC**ACCTCCACCTCCCTTGTACAGCTCGTCCATTCCT	
SIM10F	*dendra2* (F)	GCAGCTCTTCG**ATG**AACCTTATTAAGGAAGATATG	pSM12
SIM10R	*dendra2* (R)	GCAGCTCTTCG**ACC**GCCGCTTCCCCATACTTGACTTGGTAG	
SIM1F	*dendra2* (F)	GCAGCTCTTCG**GGT**GGAAGCGGCAACCTTATTAAGGAAGATATG	pSM03
SIM1R	*dendra2* (R)	GCAGCTCTTCG**TTA**CCATACTTGACTTGGTAG	
SIM2F	*mkate2* (F)	GCAGCTCTTCG**GGT**GGAGGTGGAGGTGTCTCCGAGCTCATTAAAGAAAACA	pSM04
SIM2R	*mkate2* (R)	GCAGCTCTTCG**CGT**ACCTCCACCTCCACGGTGTCCGAGCTTGGA	
SIM3F	*mkate2* (F)	GCAGCTCTTCG**ACG**GGAGGTGGAGGTGTCTCCGAGCTCATTAAAGAAAACA	pSM05
SIM3R	*mkate2* (R)	GCAGCTCTTCG**TTA**ACGGTGTCCGAGCTTGGA	
SIM6F	*mscarlet* (F)	GCAGCTCTTCG**GGT**GGAGGTGGAGGTGTCTCCAAGGGCGAGGCA	pSM08
SIM6R	*mscarlet* (R)	GCAGCTCTTCG**TTA**CTTGTACAGCTCGTCCATTCCT	
mScarlet (SAPC3)F	*mscarlet* (F)	GCAGCTCTTCG**GGT**GTCTCCAAGGGCGAGGCAGTCAT	pSDH57
mScarlet (SAPC3)R	*mscarlet* (R)	GCAGCTCTTCG**CGT**GGCCGCGGCTTTTGCAGCGG	
XF84F	*lov* (F)	GCAGCTCTTCG**ACG**CCTCGTCTTGCTGCT	pXF276
XF84R	*lov* (R)	GCAGCTCTTCG**TTA**GACCCAAGTGTCGACGGC	
XF12F	*pie-1 3′UTR* (F)	GCAGCTCTTCG**TAA**TTTTGCCGTATTTTCCAT	pXF85
XF12R	*pie-1 3′UTR* (R)	GCAGCTCTTCG**TAC**ATCATCGTTCACTTTTCAC	
tbb2 3′UTR (SAPC5)F	*tbb-2 3′UTR* (F)	GCAGCTCTTCG**TAA**ATGCAAGATCCTTTCAAGCATTC	pSDH54
tbb2 3′UTR (SAPC5)R	*tbb-2 3′UTR* (R)	GCAGCTCTTCG**TAC**GACTTTTTTCTTGGCGGCAC	
Halo (SAP C3)F	*halotag* (F)	GCAGCTCTTCG**GGT**GGAAGC	pSDH51
Halo (SAP C3)R	*halotag* (R)	GCAGCTCTTCG**CGT**TCCGGAGATCTCGAGGGTGG	
ePDZ (SAP C4)F	*epdz* (F)	GCAGCTCTTCG**ACG**GGAGGTTCCGGAGGATCTGGC	pSDH52
ePDZ (SAP C4)R	*epdz* (R)	GCAGCTCTTCG**TTA**CGTCGCGACGGGTGGAT	
unc-54 (SAPC5)F	*unc-54 3′UTR* (F)	GCAGCTGTTCG**TAA**GAGCTCCGCATCGGCCGCTG	pSDH66
unc-54 (SAPC5)R	*unc-54 3′UTR (*R)	GCAGCTCTTCG**TAC**AAACAGTTATGTTTGGTATATTGGGA	
Eg717	Replace pCFJ350 MCS (F)	TCGAGTGGCGAAGAGCCCATGGATCCCATATGGAATTCTGCAGGCCTGCTCTTCGGTAA	pXF87
Eg718	Replace pCFJ350 MCS (R)	CTAGTTACCGAAGAGCAGGCCTGCAGAATTCCATATGGGATCCATGGGCTCTTCGCCAC	
XF30F	Mutate SapI site in pCFJ350	GATTATGGGCACTTCTTTTATCC	pXF87
XF30R	Mutate SapI site in pCFJ350	CGACAAGCAACTTTTCTATAC	
XF31F	Mutate SapI site in pCFJ350	AATGGCGAAGtGCAAAGCAGAG	pXF87
XF31R	Mutate SapI site in pCFJ350	GTTTCCTGAAAATAATGTAACTTGAATTG	

Note: For the expression plasmid pJF13 annealed oligos were used to generate HSP-3(aa 1-19) in cassette 2.

Additional oligo sequences used to generate pSDH50:

TOMM-20 short forward. GCAGCTCTTCG**ATG**TCGGACACAATTCTTGGTTTCAAcaaatcaaacgtcgttttggctgctggaattgctggagccgctttcctcggctactgcatttacttcgatcataagagaatcaacgctccagactacaaggacaagattaggcaaagtcagtgttttaacaacatatttccttcggatttttatctaaaaacaacttattttctttcagagagaCGTGCCCAGGCTGGAGCAggagctggtgcaggcgctggagccggagccGGTCGAAGAGCtgc.

TOMM-20 short reverse GCAGCTCTTCG**ACC**ggctccggctccagcgcctgcaccagctccTGCTCCAGCCTGGGCACGtctctctgaaagaaaataagttgtttttagataaaaatccgaaggaaatatgttgttaaaacactgactttgcctaatcttgtccttgtagtctggagcgttgattctcttatgatcgaagtaaatgcagtagccgaggaaagcggctccagcaattccagcagccaaaacgacgtttgatttgTTGAAACCAAGAATTGTGTCCGACATCGAAGAGCtgc.

### Assembly reaction

Assembly reactions (total final volume of 50 μL) included 1 nM of pXF87 and of each donor cassette plasmid, 400 units of T4 DNA ligase (NEB), 10 units of SapI enzyme (NEB), 1x NEB CutSmart buffer and 1 mM ATP. For assemblies including annealed oligos, phosphorylated annealed oligos were used at a final concentration of 3 nM in the assembly reaction. Reactions were incubated for 22-24 hr at 25°, and transformed into Stellar Competent cells (Clontech). Four to six plasmid clones were first screened by restriction digest with XhoI and SpeI. Plasmids with the correct restriction digest pattern were sequenced across each cassette boundary. MosSCI targeting vector assembly reactions are listed in Table 4. Note that because the background of unassembled vectors in our assembly reactions was typically low, our protocol omits the counterselection restriction enzyme step described in the original SapTrap protocol (Schwartz and Jorgensen 2016).

**Table 4:** MosSCI targeting vectors used in this study

Name	Comments	Assembly									
pXF87	MosSCI backbone	Derived from pCFJ350									
		**Donor vectors used for assembly**								**Assembly efficiency**	
		**Cassettes**								**Digestion**	**Sequencing**
		**1**		**2**		**3**		**4**			
pJF13	ER lumen, Halotag	pXF121		XF17F/R*^a^*		pXF90		pXF85		4/5	2/2
pJF17	Mitochondrial OM, Halotag	pXF121		pJF7		pXF88		pXF85		4/5	1/2
pXF253	ERES + nuclear pores (NPP-20), GFP	pXF121		pXF 250		pJF6		pXF85		4/6	2/2
pXF255	ERES + nuclear pores (NPP-20), Halotag	pXF121		pXF 250		pXF88		pXF85		5/6	2/2
pXF266	Mitochondrial matrix, Halotag	pXF121		pXF 262		pXF88		pXF85		1/4	1/1
pSM20	Mitochondrial OM, mKate2	pXF121		pJF7		pXF130		pXF85		4/5	2/2
pSM22	Mitochondrial OM, mScarlet	pXF121		pJF7		pSM08		pXF85		4/5	2/2
pSM17	Mitochondrial OM, Dendra2	pXF121		pJF7		pSM03		pXF85		4/5	2/2
pSM16	Mitochondrial OM, GFP	pXF121		pJF7		pJF6		pXF85		2/5	2/2
		**1**	**2**		**3A**		**3B**		**4**		
pSDH68	Mitochondrial OM, Halotag, LOV	pXF121	pSDH50		pSDH51		PCR fragment		pSDH54	11/15	2/2

a Annealed oligos.

### Transgenesis

Double-stranded breaks at Mos1 landing sites were generated using CRISPR/Cas9. With the exception of strains EGD623, EGD629, EGD631 and EGD633, injection mixes contained 50 ng/μL of each of the following vectors: an assembled MosSCI targeting vector, pXW7.01 and pXW7.02 sgRNA/Cas9 vectors (gifts from Katya Voronina, University of Montana), which direct Cas9 to generate double-stranded breaks at the *ttTi5605* universal MosSCI insertion site. For strains EGD623, EGD629, EGD631 and EGD633, injection mixes contained 0.25 μg/μL Cas9 protein, 0.1 μg/μL tracrRNA, 0.028 μg/μL crRNA BH0278 (GCGUCUUCGUACCUUUUUGGGUUUUAGAGCUAUGCUGUUUUG), 0.028 μg/μL crRNA BH0279 (GUCCCAUCGAAGCGAAUAGGGUUUUAGAGCUAUGCUGUUUUG) (Dharmacon) and 0.1 μg/μL assembled MosSCI targeting plasmid. The universal MosSCI strains EG8078 or EG8079 (Frøkjær-Jensen *et al.* 2014) were injected, singled and incubated for 10 days at 20°. ∼10 worms from plates containing non-Unc animals were transferred to new plates. Plates that stably gave rise to non-*unc* progeny were visually screened for fluorescent transgene expression.

### HaloTag staining

20 to 30 L4 worms were stained in 25 μL S medium containing concentrated OP50 bacteria and 2.5 μM of either JF_549_ HaloTag ligand or JF_646_ HaloTag ligand (Grimm *et al.* 2015) in a darkened 96-well plate shaking at 150 rpm for 19 hr at 23°. Water was placed in the neighboring wells to help prevent evaporation. Animals were recovered on NGM plates for up to two hours before imaging.

### MitoTracker deep red staining

L4 worms were fed overnight on an NGM plate that had been seeded with 100 μL concentrated OP50 bacteria mixed with 1 μL of 1 mM MitoTracker Deep Red FM dye (Cell Signaling Technology, Cat #8778S).

### Imaging

With the exceptions of the TOMM-20::Dendra2 strain and optogenetic strains (Figure 4), all images were collected on a spinning-disk microscope built on a Nikon Eclipse Ti base and equipped with an Andor CSU-W1 two camera spinning disk module, Zyla sCMOS cameras, an Andor ILE laser module and a Nikon 100X Plan Apo 1.45 NA oil immersion objective (Micro Video Instruments, Avon, MA).

TOMM-20::Dendra2 was imaged on a Marianas spinning disk microscope (Intelligent Imaging Innovations) built around a Zeiss Axio Observer Z.1 equipped with a Photometrics Evolve EMCCD camera, 50 mW 488 and 561 nm solid state lasers, a CSU-X1 spinning disk (Yokogawa, Tokyo Japan) and a Zeiss 100X Plan-Apochromat objective. Photoconversion was performed by 5 sec illumination with a 405 epifluorescent light source.

To stimulate the relocalization of mitochondria (Figure 4), embryos were illuminated with a 50 mW 640 nm solid-state laser used to excite MitoTracker DeepRed (20% laser power, 100 msec exposure, camera gain of 1) and a 50 mW 488 nm solid-state laser used to stimulate the interaction between ePDZ and LOV domains (80% laser power and 100 msec exposure). A Plan-Apochromat 100x/1.4 NA oil immersion DIC objective (Zeiss) was used and Z-stacks (one micrometer step size, 11 steps) were collected at 60-second intervals. The images displayed in Figure 4 are maximum intensity projections of three Z planes from the cell midplane.

### Data availability

**Figure 1:**
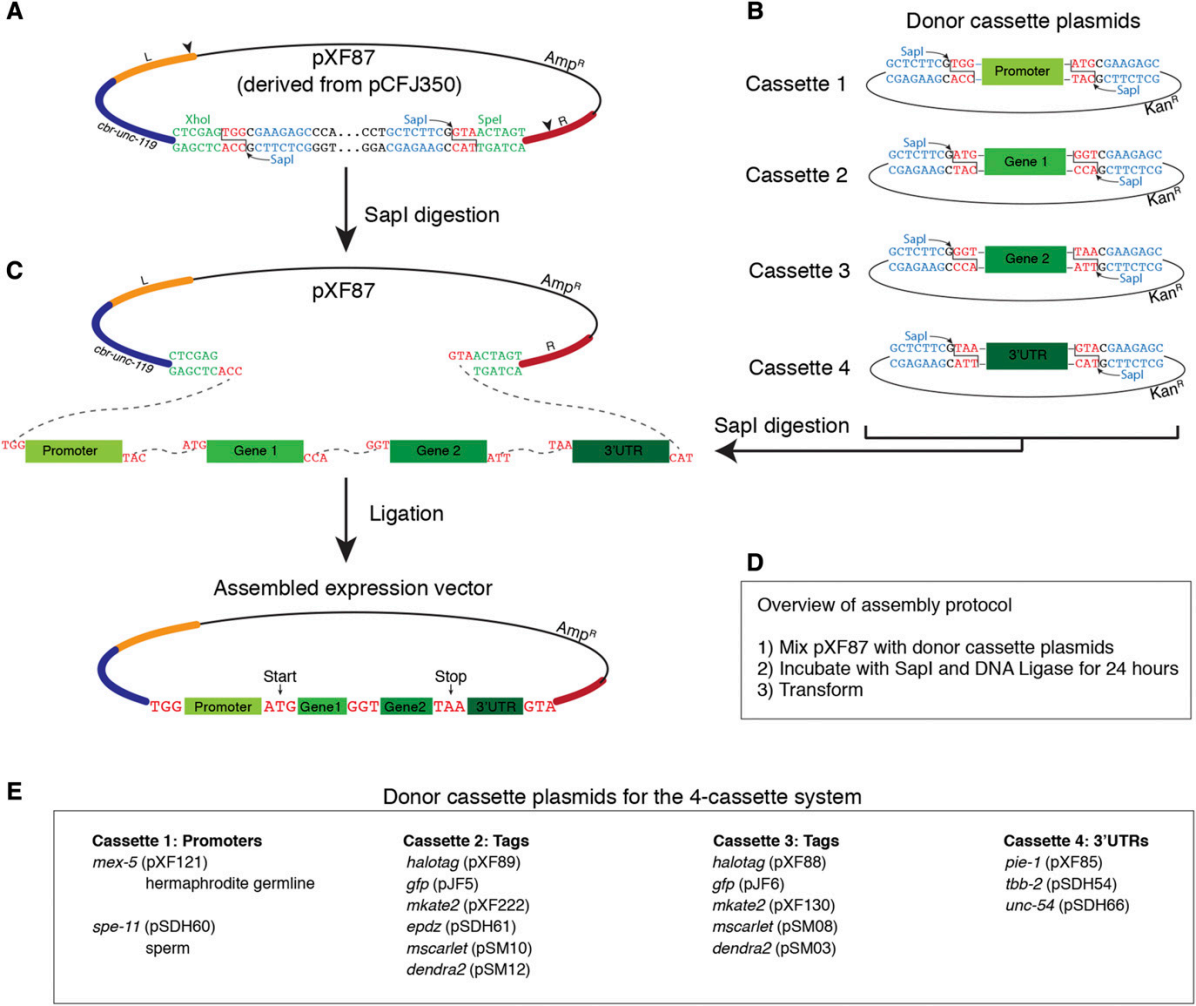
SapTrap assembly of MosSCI targeting vectors using the four-cassette system. A. The MosSCI targeting vector pXF87 was derived from pCFJ350 by mutating two SapI restriction sites (indicated by arrowheads in the “Left” (L) and “Right” (R) homology arms) and introducing two SapI sites (blue text) between the XhoI and SpeI sites (green text). SapI cleavage sites are in red text. The SapI recognition sites are oriented such that upon digestion they are removed from the vector backbone. The cbr-*unc-119* gene is used as a positive selection marker to facilitate the identification of transgenic animals. B. Design of the donor cassette vectors used for the 4-cassette cloning strategy. C. The curved dotted lines indicate the overhangs that anneal during the ligation reaction. D. Overview of the assembly protocol. For a detailed protocol, see the Materials and Methods section. E. Summary of available promoter, gene tag and 3′UTR donor cassette plasmids.

With the exception of EGD633, the *C. elegans* strains generated in this study have been deposited at the Caenorhabditis Genetics Center (CGC; https://cgc.umn.edu). The plasmids listed in Figures 1 and 3 have been deposited at Addgene (http://www.addgene.org). Other donor plasmids, assembled expression plasmids and EGD633 are available upon request. Supplemental materials describing the sequence of tag donor cassettes are available through the GSA figshare portal: https://doi.org/10.25387/g3.9978611.

## Results

### Adaptation of the SapTrap system for cloning MosSCI targeting vectors

To adapt the SapTrap approach (Schwartz and Jorgensen 2016) for the assembly of MosSCI targeting vectors, we started by making two changes to the universal MosSCI targeting vector pCFJ350 (Frøkjær-Jensen *et al.* 2012), which targets transgenes for insertion at the commonly used *ttTi5605* site (Frøkjær-Jensen *et al.* 2008). First, we introduced single base pair changes to disrupt the two SapI restriction sites located in the “Left” and “Right” homology arms of pCFJ350. Second, we inserted two SapI sites into the multiple cloning site that were oriented such that they are removed from the vector backbone by digestion with SapI. The resulting MosSCI targeting vector was named pXF87 (Figure 1A). Although pXF87 is compatible with the standard Mos1-mediated transgenesis protocol, the transgenic strains described in this study were isolated using CRISPR/Cas9 to generate double-stranded breaks in MosSCI integration sites (described in the Methods section).

We next cloned a series of plasmids that contain donor cassettes flanked by SapI restriction sites (Figure 1B). Following digestion with SapI, the cassettes are liberated from the vector backbone and are flanked by 5′ overhangs that direct their order of assembly in pXF87 (Figure 1C). A four-insert cassette system was designed with a promoter in cassette 1, gene fragments in cassettes 2 and 3 (typically a gene and a tag) and a 3′UTR in cassette 4 (Figure 1B). To minimize the inclusion of extraneous sequences, the junction between the first and second cassettes is the translation start (ATG), between second and third cassettes is glycine (GGT) and between the third and fourth cassettes is the ochre translation stop codon (TAA) (Figure 1C). Donor cassettes encoding tags (such as fluorescent proteins) include short flexible linkers at the protein fusion site (the carboxy terminus of cassette 2 and the amino terminus of cassette 3) (Supplemental Figure S1- S7). The currently available promoter, tag and 3′UTR donor cassette plasmids are listed in Figure 1E and Table 2.

The *C. elegans* germline is a notoriously difficult tissue in which to achieve stable transgene expression due to silencing of multi-copy extra-chromosomal arrays (Kelly *et al.* 1997), single-copy insertions generated by MosSCI (*e.g.*, (Shirayama *et al.* 2012; Frøkjær-Jensen *et al.* 2016)) or endogenous genes tagged using CRISPR/Cas9 gene editing (*e.g.*, (Fielmich *et al.* 2018)). Each of our tag donor cassettes encoding gene tags incorporates at least one modification that buffers against silencing, including the inclusion of PATC introns in HaloTag and ceGFP (Frøkjær-Jensen *et al.* 2016), the elimination of piRNA binding sites in mScarlet, mKate2 and Dendra2 (Seth *et al.* 2018; Zhang *et al.* 2018) and the use of sequence motifs found in native germline genes in ePDZ and the LOV domain (Fielmich *et al.* 2018).

Similar to the SapTrap method developed by Schwartz and Jorgensen (Schwartz and Jorgensen 2016), MosSCI targeting vectors were assembled in a single tube by incubating pXF87, four donor cassette plasmids, SapI enzyme, ATP and T4 DNA ligase at 25° for 22 - 24 hr (Figure 1D and Materials and Methods). This reaction was then transformed into *E. coli* and plasmid clones were screened by restriction enzyme digestion followed by sequencing. We assembled nine vectors using the 4-cassette system and 32 of 46 (69.6%) of the plasmids screened had the correct restriction digest pattern (Table 4). Of the vectors with the correct restriction digest pattern, 22 of 23 were correct based on Sanger sequencing analysis of the cassette junctions. Therefore, the SapTrap method provides an efficient method for the assembly of MosSCI targeting vectors.

### A collection of fluorescent ER and mitochondria strains

**Figure 2:**
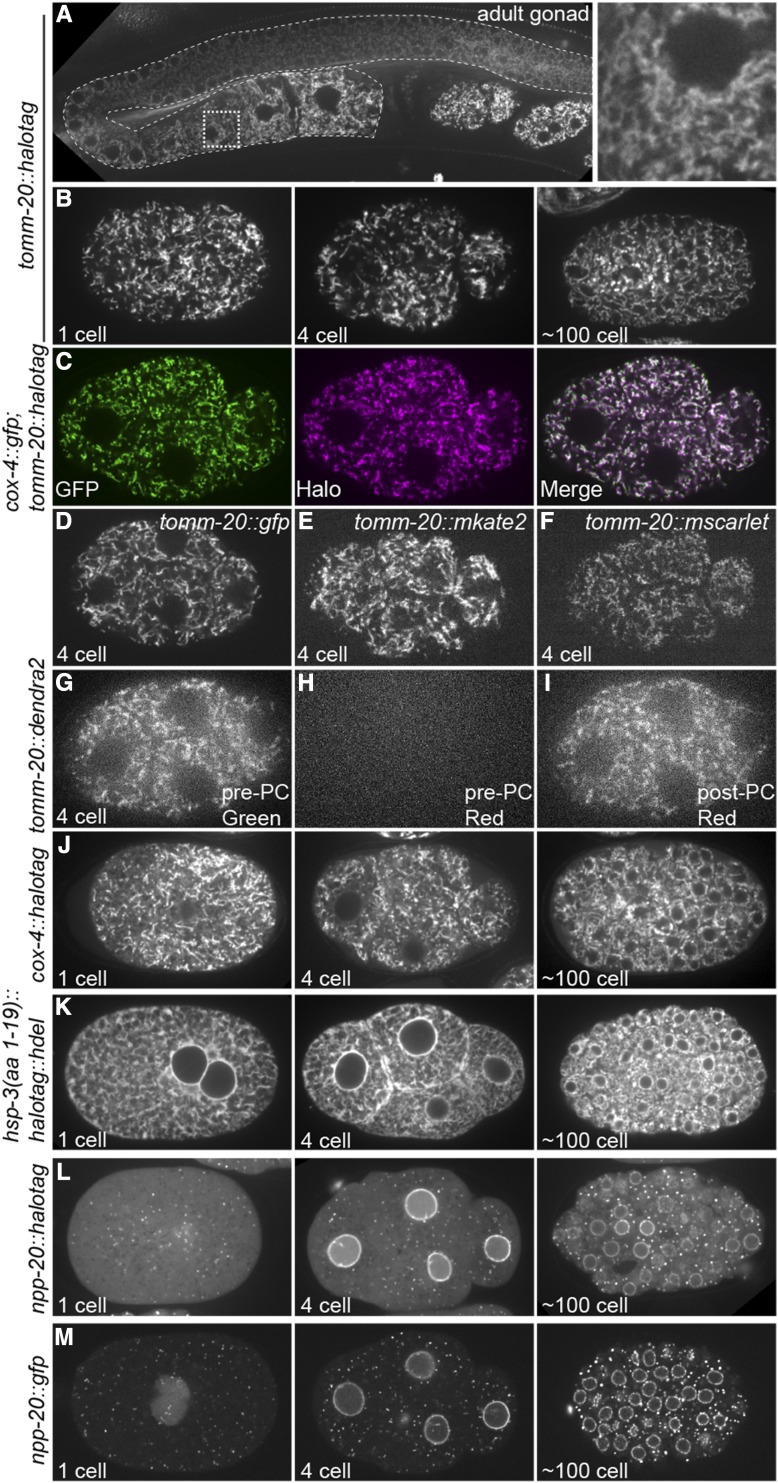
Images of transgenic strains. A. Images of TOMM-20::HaloTag labeled with JF_646_ HaloTag ligand in the adult gonad (outlined with curved dotted line), including an inset of the region in the stippled box. B. Images of embryos expressing TOMM-20::HaloTag labeled with JF_646_ HaloTag ligand at the 1-cell, 4 cell and ∼100 cell stages. C. Images of a 4 cell embryo expressing TOMM-20::HaloTag labeled with JF_646_ HaloTag ligand (magenta) and COX-4::GFP (green) (Raiders *et al.*, 2018). D – F. Images of embryos expressing the indicated transgenes at the 4-cell stage. G – I. Images of a 4 cell embryo expressing TOMM-20::Dendra2 before and after photoconversion (PC). Dendra2 switches from green to red fluorescence upon photoconversion. J – M. Images of embryos expressing the indicated transgenes at the 1-cell, 4 cell and ∼100 cell stages.

We used SapTrap-assembled MosSCI targeting vectors to generate a collection of transgenic strains for analysis of endoplasmic reticulum and mitochondrial dynamics. We first targeted GFP, mKate2, mScarlet, Dendra2 and HaloTag to the cytoplasmic face of the mitochondrial outer membrane by fusing them to the carboxy terminus of TOMM-20. The expression of these transgenes was controlled by the *mex-5* promoter and by the *pie-1* 3′UTR, which results in germline expression that increases around the bend of the adult hermaphrodite gonad (Merritt *et al.* 2008) (Figure 2A). Strains expressing TOMM-20 fused to HaloTag were labeled with the fluorescent JF_646_ HaloTag ligand (Grimm *et al.* 2015) by feeding hermaphrodites bacteria mixed with the ligand. Each TOMM-20 fusion protein exhibited the expected tubular localization pattern in the early embryo (Figure 2B-I). We confirmed that TOMM-20::HaloTag colocalized to the same organelle as the mitochondrial matrix protein COX-4::GFP (Raiders *et al.* 2018) (Figure 2C). We additionally generated strains in which the HaloTag was targeted to the mitochondrial matrix (COX-4::HaloTag) (Figure 2J) and the lumen of the endoplasmic reticulum (HSP-3(aa 1-19)::HaloTag::HDEL) (Figure 2K) (Lee *et al.* 2016). We fused both GFP and HaloTag to NPP-20, the worm homolog of SEC13, which is both a component of the COPII coat that concentrates to ER exit sites (ERES) (D’Arcangelo *et al.* 2013) and a component of nuclear pore complexes (Siniossoglou *et al.* 1996) (Figure 2L, M).

### Five-cassette system

**Figure 3:**
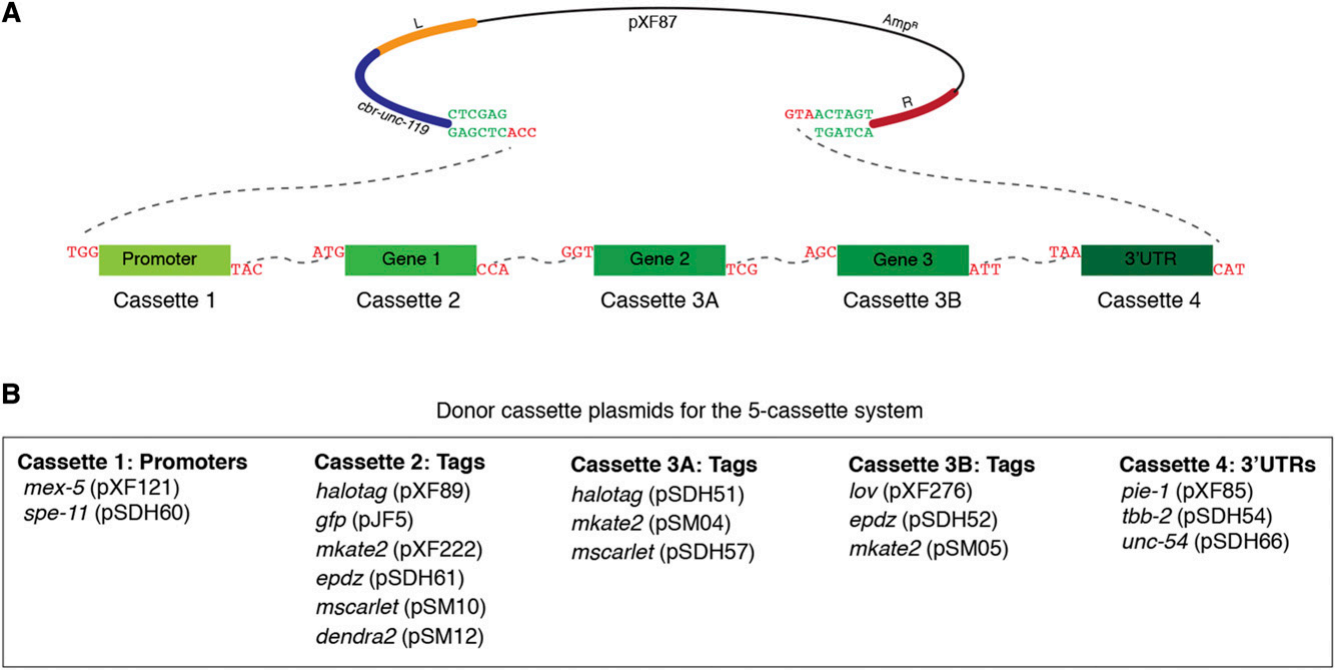
SapTrap assembly of MosSCI targeting vectors using the five-cassette system. A. Schematic of pXF87 and the donor cassettes following SapI digestion. The dotted lines indicate the overhangs that anneal during ligation. B. Summary of available promoter, gene tag and 3′UTR donor cassette plasmids for the five-cassette system.

**Figure 4:**
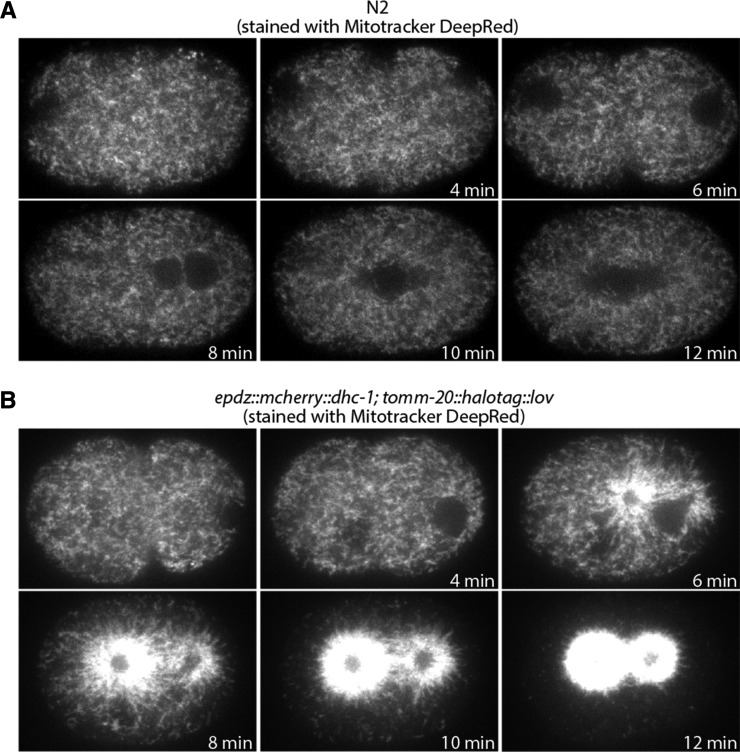
Optogenetic control of mitochondrial distribution in the 1-cell embryo. A. Control embryo stained with Mitotracker DeepRed and imaged with 488 nm and 640 nm illumination (640 nm channel shown). B. 1-cell *epdz*::*mcherry*::*dhc-1*; *tomm-20*::*halotag*::*lov* embryo stained with Mitotracker DeepRed and imaged with 488 nm and 640 nm illumination (640 nm channel shown). The 488 nm illumination was used to stimulate the interaction between the ePDZ and LOV domains.

One of the advantages of the SapTrap approach is that it can be easily expanded to include additional insert fragments to create more complex transgenes. To establish a five-cassette system, we used the cassettes 1, 2 and 4 from the four-cassette system and replaced cassette 3 with cassettes 3A and 3B (Figure 3A and 3B). We used this approach to generate an optogenetic system to control the localization of mitochondria in the early embryo based on the light induced interaction between the ePDZ and LOV domains (Strickland *et al.* 2012; Fielmich *et al.* 2018). We assembled a MosSCI targeting vector that directed expression of TOMM-20::HaloTag::LOV, which targets the LOV domain to the mitochondrial outer membrane. 11 of 15 assembled plasmids had the corrected restriction digest pattern and 2 of 2 of these plasmids were correct by Sanger sequence analysis of the cassette junctions. A TOMM-20::HaloTag::LOV strain was crossed with a strain in which the dynein heavy chain DHC-1 was fused to ePDZ (Fielmich *et al.* 2018). Whereas mitochondria in wild-type embryos are dispersed through the cytoplasm (Figure 4A), upon the recruitment of ePDZ::mCherry::DHC-1 to mitochondria by stimulation with 488 nm light, mitochondria were transported onto centrosomes, leaving the peripheral cytoplasm largely devoid of mitochondria (Figure 4B).

## Discussion

The SapTrap system described here provides an efficient and simple method for the assembly of MosSCI targeting vectors. This approach is similar to the Gateway assembly system (ThermoFisher Scientific) in that once donor cassette plasmids are cloned, they can be assembled in any modular combination. The Gateway system has been widely used to generate MosSCI transgenes and is attractive because there are large collections of promoter, ORF, and 3′UTR donor plasmids available (Brasch *et al.* 2004; Dupuy *et al.* 2004; Mangone *et al.* 2010; Zeiser *et al.* 2011). However, the Gateway system has disadvantages, including i) ∼25 bp *att* recombination sites present between each cassette after assembly, ii) the cost of proprietary enzyme mixes, and iii) the difficulty in assembling more than four cassettes together. In contrast, the SapTrap system i) uses three-base pair junctions, two of which are designed to encode the translation start and STOP codons, ii) is relatively inexpensive, and iii) can efficiently assemble at least 5 cassettes. In principle, the number of cassettes could be increased if desired. The most significant consideration in generating new donor cassette plasmids for SapTrap assembly is that internal SapI sites cannot be present within the donor cassette sequence. Gibson cloning also allows the “scar-free” cloning of transgene vectors, but the specific cloning strategies must be designed for each unique vector. While we have focused on generating transgenes expressed in the hermaphrodite germline, the MosSCI targeting vector pXF87, the gene tag donor cassettes and cloning approach described here should be readily adaptable to expressing transgenes in other tissues.

The advantages of tagging and fluorescently labeling proteins with the HaloTag include increased brightness and photostability (especially compared to red fluorescent proteins) and excellent optical pairing with green fluorescent proteins for 2-color imaging. Additionally, HaloTag labeling offers the flexibility to label a single strain with either JF_549_ HaloTag ligand or JF_646_ HaloTag ligand (Grimm *et al.* 2015). The disadvantages of HaloTag labeling include the need to introduce the fluorescent ligand (for example, using small scale liquid culture) and the cost of the ligand. Additionally, care should be taken to optimize labeling procedures for each protein to maximize labeling efficiency and minimize background from free ligand. In practice, we find that HaloTag labeling is particularly useful when photobleaching of conventional fluorescent proteins is limiting and/or when imaging in far red is advantageous.
